# Case report: A rare but fatal complication of hysteroscopy—air embolism during treatment for missed abortion

**DOI:** 10.3389/fmed.2024.1504884

**Published:** 2024-12-06

**Authors:** Yang Ou, Jun-Qiang Li, Rong Tang, Duo-Na Ma, Yang Liu

**Affiliations:** ^1^Department of Obstetrics and Gynecology, Affiliated Hospital of Southwest Jiaotong University, The Third People’s Hospital of Chengdu, Chengdu, China; ^2^Department of Obstetrics, Sichuan Jinsin Xinan Women’s & Children’s Hospital, Chengdu, China

**Keywords:** hysteroscopy, missed abortion, dilation and curettage, air embolism, case report

## Abstract

Hysteroscopic procedures complicated by air embolism (AE) are exceptionally rare occurrences in clinical practice, and there have been no previously reported cases of AE associated with hysteroscopic dilation and curettage. While the overall incidence of this complication is low, the consequences can be devastating. During early pregnancy, the unique physiological changes, such as elevated hormonal levels and increased uterine blood supply, significantly heighten the risk of AE development. Therefore, the prompt recognition of early signs and symptoms, coupled with the implementation of timely and effective interventions, are crucial to improve patient survival rates and minimize the risk of long-term sequelae. This case report presents the characteristic clinical manifestations and imaging findings associated with AE, providing a valuable clinical reference for obstetricians and gynecologists to recognize this rare yet potentially life-threatening complication of hysteroscopic procedures. Early detection and appropriate management are essential to optimize patient outcomes and prevent catastrophic consequences.

## Introduction

Dilation and curettage (D&C) is a widely employed clinical approach for managing missed abortion, characterized by a relatively low complication rate and a straightforward operational process (1, 2). However, as a blind procedure, D&C carries the risk of damaging the basal layer of the endometrium, which may subsequently lead to infertility or embryonic arrest. To protect the patient’s fertility and reduce the incidence of complications, ultrasound-guided D&C has become the standard practice (3, 4). This technique allows for indirect visualization of the uterine cavity, but it still has inherent blind spots. In contrast, hysteroscopy enables direct and clear observation of the uterine cavity, improving the precision of the surgery, preventing residual tissue, and enabling the detection of any abnormalities within the uterine cavity (5–8). As hysteroscopic techniques have become increasingly widespread, they have been gradually applied in the management of cesarean scar pregnancy and the treatment of retained products of conception (9, 10). Although hysteroscopic procedures are generally safe and effective, complications such as uterine perforation, infection, water intoxication, and air embolism (AE) may still occur (11–13). The occurrence of an AE during hysteroscopic surgery is an extremely rare but potentially life-threatening complication. The early recognition of AE signs and the implementation of timely and effective interventions are crucial for improving patient survival and reducing long-term sequelae. Herein, we present a case report of AE caused by hysteroscopic surgery during a D&C procedure for missed abortion. This is the first reported case of AE in this specific context.

## Case presentation

The patient was a 34-year-old female with a history of two previous surgical abortions. She had no significant past medical history besides the prior procedures, and no known allergies or comorbidities. On August 15, she presented to a local hospital for hysteroscopic dilation and curettage (D&C) under general anesthesia due to a missed abortion at 10 weeks of gestation.

The procedure was performed using a Shenda-brand hysteroscope (model QD-2, outer diameter: 9 mm), equipped with a cold light source for optimal visualization. A continuous flow pump system was utilized to ensure consistent uterine distension throughout the procedure. A 5% glucose solution was used as the distension medium, with an infusion rate set at 400 mL/min. A total of 1,500 mL of glucose solution was used during the procedure, and 1,450 mL was recovered, leaving a discrepancy of 50 mL. The hysteroscopic system was also fitted with an intrauterine pressure monitor to ensure stable uterine pressure.

Cervical dilation was performed prior to the procedure using mechanical dilators, expanding the cervix to approximately 9.5 mm to facilitate hysteroscope insertion. Hysteroscopy was then utilized to examine the uterine cavity and assess the position of the missed abortion. Following this, negative pressure aspiration was employed to evacuate the pregnancy. The procedure was successfully completed, and the entire process took about 15 min, with an estimated blood loss of approximately 30 mL. At 09:41, during the hysteroscopic inspection of the uterine cavity after the evacuation, the patient’s oxygen saturation suddenly dropped to 80%, and her heart rate decreased to 64 bpm. Blood pressure was unmeasurable at this point. The attending surgeon immediately halted the procedure, and anesthesia staff initiated mask ventilation with supplemental oxygen. Despite this, oxygen saturation continued to fall, reaching 70%, and the heart rate decreased further to 52 bpm.

Recognizing the signs of a possible AE, the surgical team began cardiopulmonary resuscitation (CPR) immediately. Two intravenous access lines were quickly established, and rapid fluid resuscitation was initiated. Dopamine (1 mg/mL) was administered intravenously to support blood pressure. By 09:53, the patient’s oxygen saturation had improved to 80%, with a heart rate of 149 bpm and a blood pressure of 130/79 mmHg. Blood gas analysis at this time showed a PCO_2_ of 47 mmHg and PO_2_ of 56 mmHg. CPR was discontinued as the patient’s condition stabilized.

**Figure 1:**
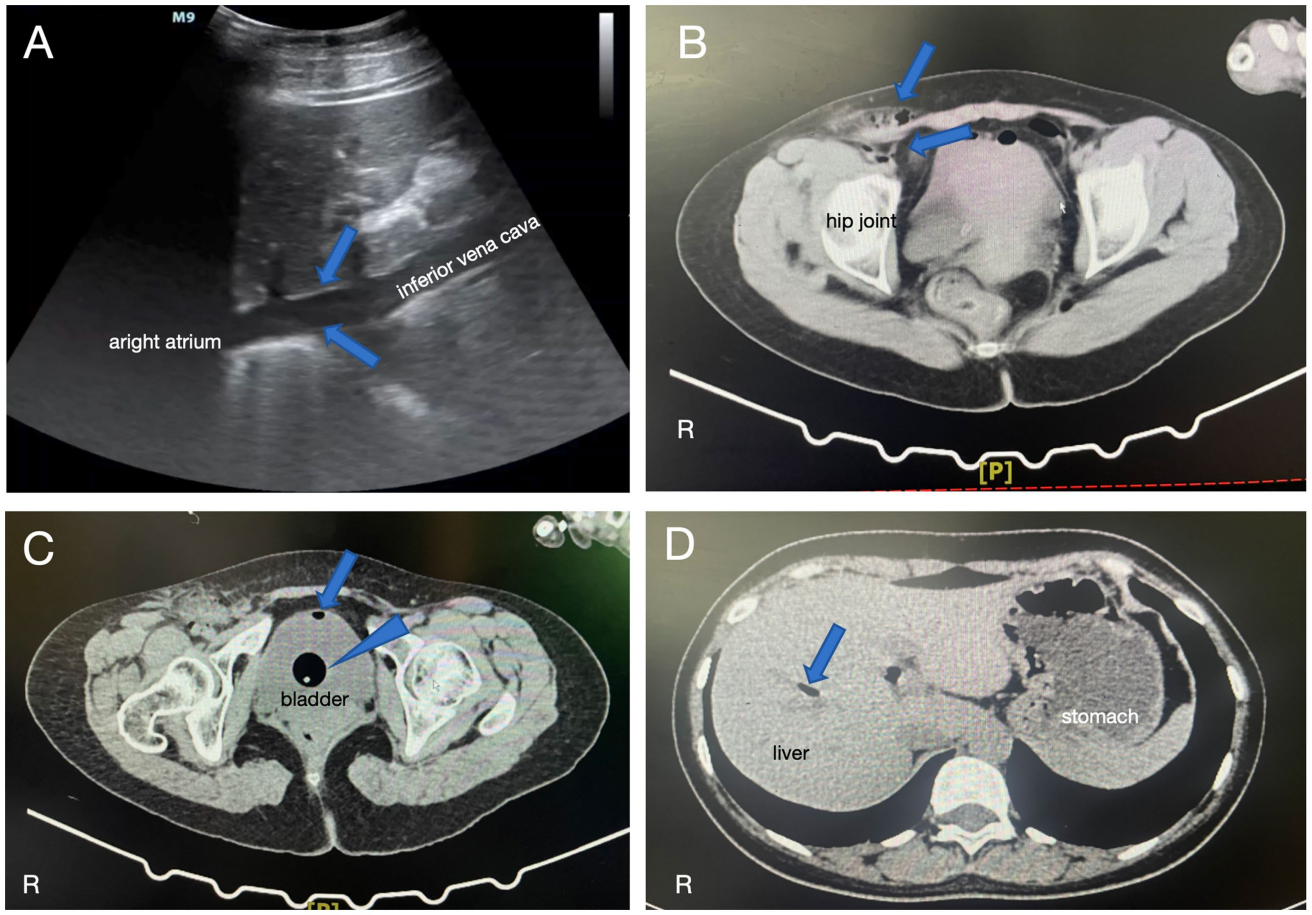
**(A)** Echocardiography showed mobile, echogenic densities in the posterior segment of the inferior vena cava, consistent with the presence of gas (blue arrows). **(B)** CT imaging revealed small amounts of gas in the right groin area and around the right iliac vessels (blue arrows). **(C)** CT showed a small amount of gas density within the bladder (blue arrow) as well as a catheter balloon shadow (blue arrowhead). **(D)** CT also demonstrated scattered gas in the intrahepatic bile duct region (blue arrow).

At 10:13, the patient was intubated, and mechanical ventilation was started with an FiO_2_ of 100% and positive end-expiratory pressure (PEEP) of 10 mmHg. Echocardiography revealed mobile, echogenic densities in the inferior vena cava below the liver, confirming the diagnosis of AE (Figure 1A). Bedside chest radiography revealed diffuse, bilateral pulmonary opacities, consistent with pulmonary edema.

The patient was managed with cefuroxime for infection prophylaxis, midodrine for vasopressor support, mannitol for dehydration, furosemide for diuresis, and ulinastatin for its anti-inflammatory properties. By 12:20, oxygen saturation had improved to 99% with mechanical ventilation, the heart rate was 121 bpm, and the blood pressure was 132/82 mmHg on a midodrine infusion at 24 mg/h.

The patient was then transferred to a tertiary referral hospital in Chengdu for further management. CT scans performed after transfer showed small amounts of gas in the right groin area and around the right iliac vessels (Figure 1B). Additionally, gas was detected in the bladder (Figure 1C) and scattered intrahepatic gas within the bile ducts (Figure 1D). Over the following days, the patient’s condition gradually improved with comprehensive care.

Follow-up imaging revealed complete resolution of the pulmonary edema, pleural effusion, and abdominal free gas. The patient was discharged on the 19th day of admission with good recovery.

## Discussion

The incidence of AE as a complication of hysteroscopic procedures is estimated to be extremely low, ranging from 0.002 to 0.01%, but the mortality rate can reach as high as 28% (12, 13). However, the risk of AE increases when hysteroscopic surgery is performed during pregnancy. This is primarily due to the elevated hormonal levels, increased uterine blood supply to the endometrium and myometrium, and the characteristic softening of the uterine muscle that occurs during pregnancy. After the removal of the gestational products and decidua, the extensive exposure of the uterine blood vessels further increases the probability of air entering the vascular system (14, 15). Therefore, when performing hysteroscopic procedures on pregnant patients, special attention must be paid to the prevention of AE. Strict intraoperative monitoring and the implementation of appropriate preventive measures are essential to mitigate this potentially life-threatening complication.

When gas emboli lead to ineffective pulmonary circulation and ventilation-perfusion mismatch, resulting in the inability to effectively eliminate carbon dioxide from the body, the initial presentation is a decrease in end-tidal carbon dioxide pressure. This is rapidly followed by a decline in blood oxygen saturation, leading to acute ischemic hypoxia in vital organs, including the coronary arteries and the brain. This cascade of events can trigger hypotension, ST-segment elevation, right bundle branch block, and even cardiorespiratory arrest (11, 15). The entry of gas into the cardiopulmonary system also triggers an inflammatory response, increasing vascular permeability in the respiratory system and resulting in pulmonary edema. Physical examination may reveal crackles and a characteristic “mill-wheel” murmur in the chest (12, 13). Echocardiography is a highly sensitive diagnostic tool for the detection of AE and can also be used to assess the impairment of cardiac function due to the entry of gas bubbles into the coronary arteries (9). This imaging modality plays a crucial role in the prompt diagnosis and management of this life-threatening complication.

Upon the occurrence of an AE, the surgical procedure should be immediately halted to prevent further entry of gas into the body (16). Prompt implementation of measures to maintain respiratory and circulatory support is crucial to ensure adequate perfusion to vital organs (17). In the event of cardiorespiratory arrest, continuous chest compressions can help to break up gas bubbles within the cardiac chambers, facilitating the expulsion of the air emboli and increasing venous return, which is a key step in the restoration of ventricular function (15). Hyperbaric oxygen therapy can play a pivotal role in preventing neurological complications associated with cerebral arterial gas embolism. This treatment modality helps to eliminate the gas and reduce the volume of the bubbles, thereby improving tissue oxygenation and mitigating the risk of ischemic damage to the brain and other vital organs (17).

Research has consistently demonstrated that prolonged surgical duration and increased blood loss are positively correlated with the risk of AE occurrence during hysteroscopic procedures (18). Therefore, the use of uterotonic agents to reduce bleeding and shorten the surgical time are important preventive measures against AE. Additionally, avoiding forceful cervical dilation, refraining from the use of gaseous distension media, preventing excessive Trendelenburg positioning, selecting low distension pressures, and minimizing repeated hysteroscope insertions are also crucial steps to mitigate the risk of air embolism (11, 16). Studies have suggested that nitrous oxide has a high blood-gas partition coefficient, making it readily absorbed into the bloodstream. Consequently, it is recommended to avoid the use of nitrous oxide during general anesthesia for hysteroscopic procedures (11). Furthermore, Van Dijck et al. (19) has reported that patients who developed AE had significantly shorter recovery times in the postanesthesia care unit than those without AE, underscoring the importance of extending the observation period in the postanesthesia care unit for patients undergoing hysteroscopic surgery.

Although Catena et al. (20) argued that routine preoperative laboratory testing does not reduce the incidence of postoperative complications in minor gynecologic procedures, other studies have suggested that elevated serum levels of the beta subunit of human chorionic gonadotropin, secreted by syncytiotrophoblast cells in early pregnancy, are associated with a richer blood supply around the gestational product (15). This, in turn, may increase the incidence of AE during hysteroscopic procedures.

In this case, the AE may have resulted from the combined effects of the hysteroscopic procedure and D&C. The use of a hysteroscope with continuous irrigation introduces a potential risk of air entry into the vascular system, particularly in the presence of exposed uterine blood vessels after the evacuation of gestational products. Concurrently, the D&C procedure itself may have heightened this risk by causing trauma to the uterine lining and blood vessels. The interplay between the two procedures likely contributed to the development of the AE in this case.

## Conclusion

In summary, AE is a rare but potentially life-threatening complication that can occur during hysteroscopic procedures. Throughout the surgical process, continuous monitoring of end-tidal carbon dioxide and blood oxygen saturation is crucial for the early detection and prompt management of this serious event. Immediate recognition of the signs and symptoms of AE, coupled with the implementation of appropriate resuscitative measures and supportive care, are essential to optimize patient outcomes and minimize the risk of devastating complications.

## Data Availability

The original contributions presented in the study are included in the article/supplementary material, further inquiries can be directed to the corresponding author.
